# Insights Into Natural Genetic Resistance to Rice Yellow Mottle Virus and Implications on Breeding for Durable Resistance

**DOI:** 10.3389/fpls.2021.671355

**Published:** 2021-06-29

**Authors:** Patrick J. Odongo, Geoffrey Onaga, Oliver Ricardo, Keiko T. Natsuaki, Titus Alicai, Koen Geuten

**Affiliations:** ^1^Molecular Biotechnology of Plants and Micro-Organisms, Institute of Botany and Microbiology, KU Leuven, Leuven, Belgium; ^2^National Crops Resources Research Institute, National Agriculture Research Organization, Kampala, Uganda; ^3^M’bé Research Station, Africa Rice Center (AfricaRice), Bouaké, Côte d’Ivoire; ^4^Breeding Innovations Platform, International Rice Research Institute, Metro Manila, Philippines; ^5^Graduate School of Agriculture, Tokyo University of Agriculture, Tokyo, Japan

**Keywords:** RYMV, rice-RYMV interaction, resistance mechanisms, durable resistance, rice improvement

## Abstract

Rice is the main food crop for people in low- and lower-middle-income countries in Asia and sub-Saharan Africa (SSA). Since 1982, there has been a significant increase in the demand for rice in SSA, and its growing importance is reflected in the national strategic food security plans of several countries in the region. However, several abiotic and biotic factors undermine efforts to meet this demand. Rice yellow mottle virus (RYMV) caused by *Solemoviridae* is a major biotic factor affecting rice production and continues to be an important pathogen in SSA. To date, six pathogenic strains have been reported. RYMV infects rice plants through wounds and rice feeding vectors. Once inside the plant cells, viral genome-linked protein is required to bind to the rice translation initiation factor [eIF(iso)4G1] for a compatible interaction. The development of resistant cultivars that can interrupt this interaction is the most effective method to manage this disease. Three resistance genes are recognized to limit RYMV virulence in rice, some of which have nonsynonymous single mutations or short deletions in the core domain of eIF(iso)4G1 that impair viral host interaction. However, deployment of these resistance genes using conventional methods has proved slow and tedious. Molecular approaches are expected to be an alternative to facilitate gene introgression and/or pyramiding and rapid deployment of these resistance genes into elite cultivars. In this review, we summarize the knowledge on molecular genetics of RYMV-rice interaction, with emphasis on host plant resistance. In addition, we provide strategies for sustainable utilization of the novel resistant sources. This knowledge is expected to guide breeding programs in the development and deployment of RYMV resistant rice varieties.

## Introduction

Food security is a primary concern for many economies around the world because the human population is increasing, and food production must increase to keep pace (Food and Agriculture Organization of the United Nations, 2010; Boyd et al., 2013; Savary et al., 2019). According to Valin et al. (2014), a 74% increase in food demand is expected by 2050, which would affect consumers worldwide. Meeting this demand is a daunting task, and will require major improvements in agricultural production systems, including improved strategies to minimize crop losses to biotic and abiotic stresses (Das and Rao, 2015). Cereal crops account for more than 60% of food intake and will continue to contribute significantly to global food security. This contribution is reflected in projected increases in the consumption of wheat, corn, and rice by an average of 53, 106, and 47%, respectively, by 2050 (Valin et al., 2014; FAOSTAT, 2018). Of these three main crops, rice is the most important food crop for people in low- and lower-middle-income countries. In sub-Saharan Africa (SSA), where most of the population falls within the category of low incomes, the demand for rice has increased considerably since 1982. This increased demand is due to improving economies, rising household incomes, and growing population and urbanization that have led to increased consumption of rice as a major staple (Balasubramanian et al., 2007). To meet the increased demand, the region is continuously pushing for coordinated efforts to increase rice self-sufficiency in several rice-producing countries (Seck et al., 2013). However, the prospects for rice self-sufficiency are being subverted by abiotic and biotic factors, including plant diseases. Several important rice diseases have emerged recently in SSA (Séré et al., 2013). The most notorious of these has been Rice yellow mottle virus (RYMV), which is restricted to only Africa (Bakker, 1974; Kouassi et al., 2005; Savary et al., 2019).

RYMV was first identified in East Africa in 1966 (Bakker, 1974), from where it is believed to have spatially spread westward into West Africa (Pinel et al., 2000). The virus has now spread to all rice-growing countries in the region, and as such is described as an emerging disease (Fargette et al., 2006). The natural hosts of RYMV are limited to *Oryza sativa*, *Oryza glaberrima*, and wild rice, including *Oryza logistiminata* and *Oryza barthi* (Bakker, 1970; Allarangaye et al., 2007). Recently, some wild accessions from the primary gene pool (AA genome), including *Oryza glumaepatula*, *Oryza breviligulata*, *Oryza meridionalis*, *Oryza rufipogon*, and *Oryza nivara*, have also shown susceptibility in screening experiments (Allarangaye et al., 2007; Odongo et al., 2019), which indicate that RYMV is capable of infecting several species of *Oryzae*. Clear symptoms of RYMV include severe leaf mottling, yellow-green streaking, decreased tillering, and stunting of plants during the vegetative stage. Reproductive stage symptoms include poor emergence of panicles and panicle sterility (Bakker, 1970). RYMV yield losses range from 10 to 100%, depending on the time of infection, ecology, viral strain, and the rice genotype. Plants that display severe symptoms during seedling and early vegetative stages often result in plant death (Bakker, 1974; Kouassi et al., 2005). In lowland ecologies, susceptible genotypes can be severely damaged in a short period of time during periods of intense disease activity. This has been observed in susceptible varieties grown in West African countries. For example, in Nigeria and Côte d’Ivoire, yield losses above of 90% have been reported in susceptible cultivars, Bouake 189 and FARO 29 (Onwughalu et al., 2011; Soko et al., 2016), while in Sierra Leone, losses of 82% were reported on varieties PN 623-3, TOX 516-12-SLR, and ROK 3 (Taylor et al., 1990). Most recently in Burkina Faso, losses of 84% were recorded on popular varieties, FKR56N, FKR62N, and TS2 (Traoré et al., 2015).

The prevalence of RYMV varies between rice-producing countries, probably because of multiple factors including transitions from subsistence production to large-scale intensive rice production, and the associated increase in the diversity of vectors involved in virus transmission. For example, the incidence and severity of RYMV in most of Uganda’s rice-growing areas is between 50 and 75% under rainfed lowlands (Ochola and Tusiime, 2010), while in Burkina Faso, the incidence of the disease tends to be lower at 28% (Traoré et al., 2015). In Côte d’Ivoire, the incidence is higher during the dry season than during the rainy season while the opposite is the case in Nigeria (Heinrichs et al., 1997). In Niger, RYMV is generally sporadic and field infections are often found in patches (Sarra and Peters, 2003). High incidence of RYMV has been also reported in Burundi, Zimbabwe, Ethiopia, the Central African Republic, the Democratic Republic of Congo, and Zanzibar (Traoré et al., 2001; Abubakar et al., 2003; Hubert et al., 2013). The spread and severity of the disease in the Democratic Republic of Congo is attributed to changing agricultural patterns with most farmers increasingly cultivating rice in irrigated lowlands. Such changes create a conducive environment for RYMV vectors to multiply (Hubert et al., 2013), a trend that could increase transmission and create a risk of severe infections.

The response from the scientific community to the RYMV threat has been positive and has made progress. Yield losses have been addressed through adapting RYMV control measures to local situations. This includes crop improvement toward local production systems and utilization of specific agronomic practices including the use of varietal mixtures and pesticide sprays, early planting, destruction of previous plants, ratoons and volunteer crops, removal of infected plants, crop rotation, and optimum fertilizer application. These practices aim at disrupting the life cycle of the disease and improving crop health (Traoré et al., 2009). However, their use is limited and still ineffective, especially when the disease occurs in epidemic proportions. Genetic resistance is the best feasible option for economical and sustainable long-term RYMV management. Recent advances in genetics and molecular biology have contributed to the identification of RYMV resistance genes and quantitative trait loci (QTLs) that have been mapped and some cloned from *O. sativa* and *O. glaberrima* (Ndjiondjop et al., 1999; Ioannidou et al., 2003; Albar et al., 2006; Rakotomalala et al., 2008; Thiémélé et al., 2010; Pidon et al., 2017). However, Traoré et al. (2006b) and Fargette et al. (2008a) have highlighted the nuisance associated with RYMV resistance management. The virus evolves rapidly, and resistance-breaking variants have been observed across SSA (Traoré et al., 2006a; Fargette et al., 2008a,b). So much work remains to be done to identify the best strategies to limit the prevalence and yield losses caused by RYMV. In this review, we present an update of RYMV genetics in relation to virus-rice interactions and discuss some interventions focusing on natural genetic resistance. In addition, we highlight the implications of using known genetic resistance in breeding for durable resistance to the virus in SSA.

## RYMV Genome Structure, Gene Function and Diversity

**Figure 1:**
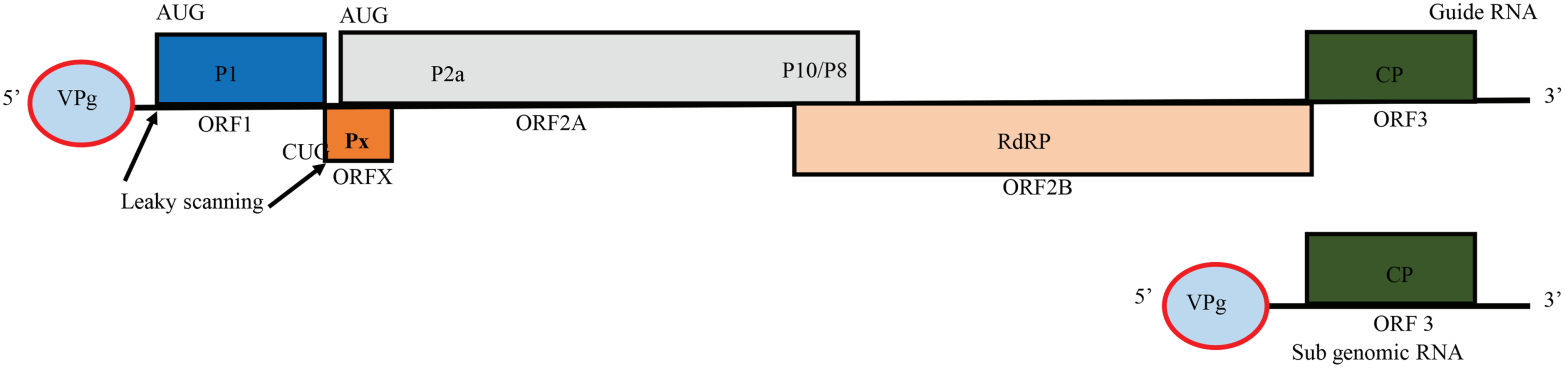
Genome organization of Rice yellow mottle virus (RYMV). Boxes represent open reading frames (ORFs) and the encoded proteins in each ORF. VPg, viral genome-linked protein; P1, gene silencing suppressor protein; P2a, viral protease; P10/P8, polyproteins with no assigned function; RdRP, RNA dependent RNA polymerase; CP, major coat protein. The figure was adapted from Ling et al. (2013).

RYMV is a member of the genus *Sobemovirus* in the *Solemoviridae* family (Bakker, 1974; Hébrard et al., 2021). The virus capsid consists of 180 copies of 26 kDa coat protein (CP) subunits assembled in a T = 3 icosahedral structure with a positive-sense single-stranded RNA (+ssRNA) particle size of 25–28 nm in diameter (Bakker, 1974; Opalka et al., 2000; Qu et al., 2000). RYMV encodes a small genome of approximately 4,500 nucleotides, of these, roughly, 330 nucleotides are in non-coding regions (Yassi et al., 1994) (Figure 1). Additionally, the virus encapsidates a non-coding viroid like satellite RNA (satRNA) of about 220 nucleotides, which depends on a helper virus for replication. The satRNA plays no role during infection process (Tamm and Truve, 2000; Sõmera et al., 2015; Hébrard et al., 2021). The genome is a polycistronic RNA harboring five overlapping open reading frames (ORFs): ORF1, ORFx, ORF2a, ORF2b, and ORF3 (Yassi et al., 1994; Ling et al., 2013). ORF1 and ORF3 are more variable while ORF2a and ORF2b are quite conserved. Because of this, the genome size may vary depending on nucleotide diversity of ORFs. For instance, the Mali strain is about 4,450 nucleotides, and some Nigerian isolates are reported to be about 4,452 nucleotides. In ORF3, a few insertion-deletions involving basic amino acids occur depending on the strain (Fargette et al., 2004). Moreover, two non-coding regions of about 80 and 289 nucleotides, respectively, are adjacent to the furthest 5' and 3' ends of the genome (Yassi et al., 1994; Hébrard et al., 2008a). ORF1 is at the 5' end, and encodes the 17.8 kDa P1 protein of 157 amino acids. This protein is involved in viral accumulation and post-transcriptional gene silencing by suppressing the mechanisms of host RNA silencing (Siré et al., 2008; Lacombe et al., 2010). P1 is dispensable for the local movement and replication of RYMV but is essential for systemic infection (Brugidou et al., 1995; Bonneau et al., 1998; Nummert et al., 2017). At the 5' terminus of ORF1 is a viral genome-linked protein (VPg) instead of a cap while the 3' end of the viral genome is not polyadenylated (Hull, 1977; Yassi et al., 1994). ORFx, a newly assigned ORF, is embedded beneath the 5' end of ORF2. It encodes a replication protein, named protein X. Unlike in other *Sobemoviruses*, ORFx does not overlap with ORF1. Its translation initiates at a highly conserved CUG codon and proceeds through a leaky scanning and ribosomal frameshift mechanism (Ling et al., 2013). The next ORFs are the two overlapping ORF2a and ORF2b. ORF2a expresses VPg in the first 134 amino acids (Yassi et al., 1994; Fargette et al., 2004), which is pinpointed to influence the virulence against resistance conferred by two major genes (*RYMV1* and *RYMV2*) (Hébrard et al., 2008b; Pinel-Galzi et al., 2016). ORF2a also encodes a polyprotein protease P2a, and two additional proteins, P10 and P8, the functions of which have yet to be investigated. ORF2b encodes the (P2b) RNA-dependent RNA polymerase (RdRP) protein. ORF3 encodes the viral coat protein (CP) translated from the sub genomic RNA. The coat protein is responsible for cell-to-cell movement, virus packaging, and stability (Yassi et al., 1994; Bonneau et al., 1998; Opalka et al., 1998).

RYMV has a distinct geographic diversity (Fargette et al., 2004, 2008b), and the highest diversity is established in East Africa–the putative center of ancestry and diversification (Pinel-Galzi et al., 2015). Five serotypes (Ser1–Ser5) across Africa are distinguished based on serological typing (Konate et al., 1997; N’guessan et al., 2000). Molecular typing of the CP of these serotypes recognizes six strains including S1, S2, and S3 in West Africa and S4, S5, and S6 in East Africa. Nucleotide and amino-acid difference between East African and West African strains can be up to 11%; however, at the nucleotide level, low variation within strain and within isolate is found (Pinel et al., 2000). Polymorphisms of amino-acids in the bipartite nuclear focusing motif of the R domain of the CP, and near the conserved position 151–154 of the S domain presumably determine differences and aggressiveness among the strains and isolates (Pinel et al., 2000). For instance, new Ugandan isolates were serotyped and grouped into Ser 4, based on polymorphisms in the amino acid sequences of CP gene (Uke et al., 2016). Recently, other variants in the S4, S6, and S1 subtypes have been identified (Adego et al., 2018). These serotypes are possibly segregated by polymorphisms in two amino acids: alanine vs threonine at position 115 and valine vs threonine at 191. These positions possibly localize in the antigenic sites and share an epitope placed within a conserved region. Thus, these polymorphisms may elucidate the cross-reactivity between RYMV isolates (Fargette et al., 2002a).

## RYMV-Host Plant Interaction

RYMV is transmitted by about 12 insect vectors, which accelerate viral spread in most localities (Sere et al., 2008; Koudamilore et al., 2014). *Chrysomelid* beetles are the primary vectors that transmit the virus from its reservoirs in a semi-persistent manner (Bakker, 1970; Abo et al., 2000; Allarangaye et al., 2006). The virus can be found in the seed but no direct seed transmission has been definitively established in host plants (Konate et al., 2001; Allarangaye et al., 2006). It is unclear whether this is due to endogenous viral elements that have been reported to constitute footprints of previous infections by existing or ancient viruses (Maumus et al., 2014). Experimentally, RYMV is mechanically transmissible through sap inoculation. Other secondary forms of RYMV transmission include farming implements, wind, irrigation water, and animals (Traoré et al., 2009). Seedbed nurseries and infected crop stubble also serve as auxiliary sources of the virus (Traoré et al., 2006b; Uke et al., 2014).

**Figure 2:**
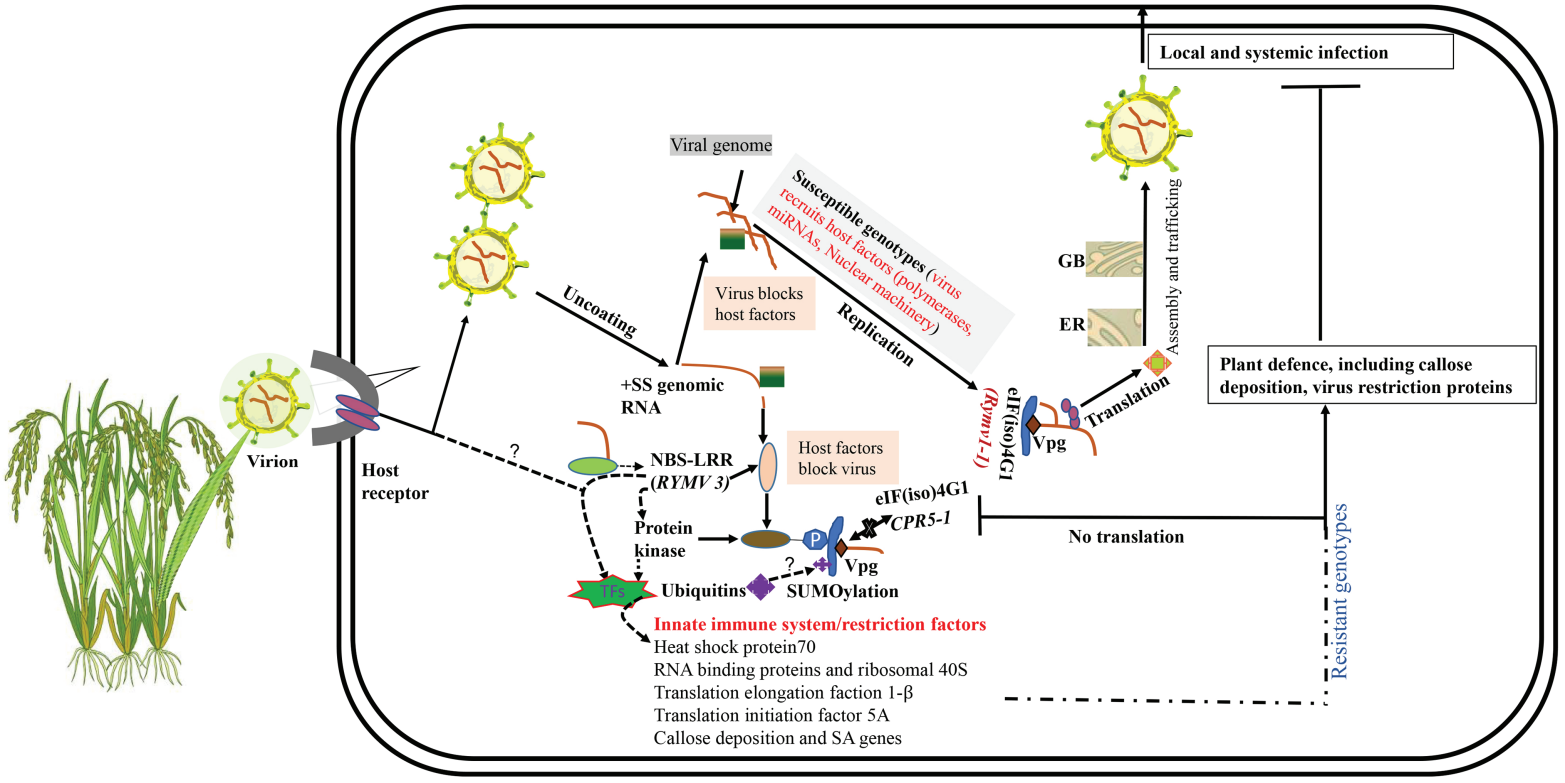
Mechanisms of rice-RYMV interactions. Viruses rely on numerous interactions with the host cell to replicate and transmit. GB, Golgi body; ER, endoplasmic reticulum. Unknown hypotheses are indicated as “?.” In a susceptible interaction, a virus attaches, un-coats, replicates, and expresses proteins, and then assembles and egresses. eIF(iso)4G1 encoded by *rymv1-1* (susceptibility allele) possesses altered sites that facilitates viral RNA Vpg binding for successful translation of viral proteins. In a resistant reaction, we hypothesize that when the virus enters the plant cell, viral RNAs or its associated molecules in the cytoplasm are recognized by NBS-LRR or unknown receptors. The signal is transmitted from NBS-LRR *via* transcription factors (TFs) to the nucleus where induced resistance genes that limit viral replication, translation, and movement are transcribed. NBS-LRR proteins could be activated by direct recognition of the viral RNA. The signal could also be transmitted *via* either protein kinase monomers or dimers upon binding to viral RNA, which phosphorylates eIF(iso)4G1, thereby leading to translation suppression. Ubiquitination and SUMOylation could also play a role in translation regulation of eIF(iso)4G1.

Viral entry is facilitated through natural wounds caused by insect vectors and/or intercellular transfer as virion or viral ribonucleoproteins (Schoelz et al., 2011; Wang et al., 2015; Garcia-Ruiz, 2018). For compatible interaction, RYMV, like all plant viruses, must achieve both local and systemic infection in their hosts (van Loon, 1987). This infection process involves a sophisticated molecular interaction between the plant host factors and virus proteins (Figure 2). Compatible host proteins facilitate encapsidation, replication, translation, movement, and assembly. After the viral (+) RNA is released in the cytoplasm, replication is initiated. Like other *Sobemoviruses*, the mechanism of replication initiation of plus-and minus-strands in RYMV is still ambiguous, and needs to be further investigated (Tamm and Truve, 2000). However, recent studies suggest that the viral replication complex (VRC) replicates the nascent complementary negative sense RNA (−) using the primary +ssRNA. Subsequent (−) RNAs undergo translation and replication cycles yielding more (+) mRNAs. These are then used to produce new viral proteins and more (−) RNA and finally, the genome is enscapsidated to produce new virus particles (Souza, 2020). The new viral particles are transported cell-to-cell *via* the plasmodesmata to the vascular tissues for systemic spread (Mandadi and Scholthof, 2013; Heinlein, 2015). The systemic spread of RYMV occurs through xylem vessels in which large amounts of viral RNA accumulate and are then transported with solutes to new cells. RYMV disrupts the pit membranes in the xylem vessels, thus enabling the movement of virus particles into new cells (Opalka et al., 1998). To facilitate the active passage of virus particles to neighboring cells, the plasmodesmata must be modified with the help of host proteins. ORF1 and ORF3 play a key role in the modification of plasmodesmata (Bonneau et al., 1998; Nummert et al., 2017). Generally, two hypotheses explain plasmodesmata modification during viral transport (Schoelz et al., 2011; Reagan and Burch-Smith, 2020): the size exclusion limit and the removal of desmotubules and endoplasmic reticulum membranes (Schoelz et al., 2011; Reagan and Burch-Smith, 2020). RYMV uses the size exclusion limit strategy for movement to new cells (Opalka et al., 1998; Kouassi et al., 2006). RYMV particles can be localized in various plant cells, such as epidermis, nucleus vacuole, mesophyll, vesicles, bundle sheath, and vascular parenchymal cells, within 3–6 days post-infection (dpi; Opalka et al., 1998; Ndjiondjop et al., 2001), as well as in the chloroplast (Brugidou et al., 2002). Intracellularly, these viral particles occur in three isoforms described based on the presence of divalent ions and pH. The first is a stable compact form that is pH independent and has Ca^2+^, the second is the stable transitional form dependent on acidic pH but devoid of Ca^2+^, and the third is the unstable swollen form dependent on basic pH and lacks Ca^2+^ (Opalka et al., 1998; Brugidou et al., 2002). The viral particle stability depends on the stage of infection in the host, for instance, the transitional and swollen isoforms are more abundant during early infection, while the compact isoforms increase during late stages of infection (Brugidou et al., 2002).

In plant cells, the virus triggers host defense reactions that involve differential expression of genes that are regulated by various signaling pathways (Whitham et al., 2006). Transcriptomic studies using ESTs and cDNA-AFLP revealed differential activation of defense, metabolic, and photosynthesis pathways (Ventelon-Debout et al., 2003, 2008). In the partially resistant cultivar Azucena, in comparison with the susceptible IR64, RYMV induces expression of defense and stress related genes. Proteomic studies, using 2D-DIGE and LC-MS/MS to compare infected cell suspensions of the resistant cultivar IR64 with those of the partially resistant cultivar Azucena, further revealed protein profiles involved in metabolism, defense, and stress-related protein translation and synthesis (Ventelon-Debout et al., 2004). Delalande et al. (2005) identified three candidate proteins belonging to multigenic families including a phenylalanine ammonia-lyase, a mitochondrial chaperonin-60, and an aldolase C; however, the role of these proteins in RYMV infection remains to be validated. Further studies by Brizard et al. (2006), using SDS-PAGE and nano-LC-MS/MS identified 223 differentially regulated proteins that were categorized into functional pathways comparable to those observed using 2D-DIGE. In an incompatible interaction, RYMV induces proteins in glycolysis pathway, defense-related proteins including superoxide dismutase (SOD) (Ventelon-Debout et al., 2004; Brizard et al., 2006). In contrast, there is a marked downregulation of SOD in susceptible cultivars (Ventelon-Debout et al., 2004). In addition, studies also reveal that there is an increased accumulation of HSP 70 in the susceptible cultivar IR64 during early RYMV stress compared to partially resistant Azucena (Ventelon-Debout et al., 2004). Expression of heat shock protein 70 (HSP70) is a common incidence in response to plant viral infections, though the function of HSP70 during viral infection is poorly understood (Aparicio et al., 2005). RYMV also induces translation and protein synthesis like ribosomal proteins, translation initiation, and elongation factors, protein disulfide isomerases, chaperone proteins, and proteins involved in protein turnover such as the 20S proteasome (Ventelon-Debout et al., 2004; Brizard et al., 2006). The role of these proteins during RYMV infection remains to be validated functionally.

While these studies provide a good view of the genes and proteins expressed during RYMV-rice interactions, no recent high throughput genome-based technologies, such as RNA-seq, have been used to study RYMV-rice interactions. To clarify the different transcriptomic responses between compatible and incompatible RYMV-rice interactions and to describe more genes involved in this process, RNA-seq based approaches ought to be applied.

## Genetic Determinants and Mechanisms of Qualitative Resistance to RYMV

Qualitative disease resistance genes show monogenic or near complete resistance and are thus called major genes (Nelson et al., 2018). Qualitative disease resistance can be described based on two models: gene-for-gene and the matching allele model (Fraile and García-Arenal, 2010). Gene-for-gene resistance involves activation of resistance proteins such as nucleotide-binding domain leucine-rich repeat proteins (NB-LRR), which play a role in the perception of pathogen invasion and activation of plant defense mechanisms. Resistant plants usually encode NB-LRR proteins which are normally genetically dominant (R genes) (Fraile and García-Arenal, 2010; Nelson et al., 2018) (Figure 2). The matching allele interaction/resistance is conferred by the absence of host factors (susceptibility factors) required for completion of the virus infection cycle (Moffett, 2009; Fraile and García-Arenal, 2010; Wang and Krishnaswamy, 2012). Some of the genes expressed in the matching allele model induce recessive resistance. Examples of recessive host factors include the eukaryotic-translation initiation factors (eIFs) such as eIF4E and eIF4G and their isoforms. Recessive resistance is the predominant form of resistance against plant viruses (Wang and Krishnaswamy, 2012; Hashimoto et al., 2016), and can be as a result of loss-of-function of susceptibility (*S* genes) (Wang and Krishnaswamy, 2012; Sanfaçon, 2015; Hashimoto et al., 2016; Garcia-Ruiz, 2018). Most of the genes conferring resistance against RYMV fall in this category. These include *RYMV1* (*Resistance* to the *yellow mottle virus1*) and *RYMV2* (Albar et al., 2006; Thiémélé et al., 2010; Orjuela et al., 2013; Pidon et al., 2017, 2020).

The first major gene to be found and most studied is *RYMV1*. *RYMV1* located on chromosome four and encodes eIF(iso)4G1. In a pro-viral interaction, eIF(iso)4G is recruited and directly interacts with VPg of RYMV for successful infection (Albar et al., 2006). In antiviral interactions, nonsynonymous mutations in eIF(iso)4G interfere with direct interaction with the VPg causing a resistant phenotype. These mutations may have occurred independently in *O. sativa* and *O. glaberrima*, each affecting the specificity of the allele/isolate interaction (Hébrard et al., 2010). The *RYMV1* locus harbors four alleles with point mutation or small deletions; including *rymv1-2*, the only allele identified in *O. sativa*, and *rymv1-3*, *rymv1-4*, and *rymv1-5*, the three alleles reported in *O. glaberrima* (Albar et al., 2006; Thiémélé et al., 2010). The resistance conferred by *rymv1-2* and *rymv1-4* is due to a substitution of a glutamic acid (E) for a lysine (K) at amino acid positions 309 and 321 of eIF(iso)4G, whereas, the resistance conferred by *rymv1-3* is characterized by a deletion of three amino acids at positions 322–324 in the central domain of eIF(iso)4G (Albar et al., 2006). A deletion in positions 313–315 in the central domain of eIF(iso)4G is responsible for the resistance conferred by *rymv1-5* (Thiémélé et al., 2010). Some rice accessions harboring *RYMV1* resistance exhibit complete resistance, and these are generally described as highly resistant accessions (Table 1).

**Table 1:** List of the resistant accessions and details of the resistance genes and their alleles.

Gene	Host factor	Allele	Viral factor	RB^1^ isolate	Source of resistance	Reference
*RYMV1*	eIF(iso)4G	*rymv1-2*	VPg	S2, S4	Gigante, Bekarosaka	Albar et al., 2006; Rakotomalala et al., 2008
		*rymv 1-3*		S4, S5, S6	Tog5681	Albar et al., 2006
		*rymv1-4*		S1, S2	Tog5463, Tog5672, Tog5438	Albar et al., 2006; Thiémélé et al., 2010
		*rymv1-5*		S2	Tog5674	Thiémélé et al., 2010
*RYMV2*	*CPR5*	*rymv2-R1*	VPg	S1, S2, S3	Tog6220, Tog6698, Tog7206, Tog7235, Tog10434, Tog7291, Tog7202,	Thiémélé et al., 2010; Orjuela et al., 2013; Pidon et al., 2020
		*rymv2-R2*			RAM 131, 1LG104, 3LG1178	Pidon et al., 2020
		*rymv2-R3*			Tog7456	
		*rymv2-R4*			Tog14367, Tog13943, Tog13709, Tog12401, Tog7197	
		*rymv2-R5*			SG32	
		*rymv2-R6*			Tog14361	
*RYMV3*	LRR	*NIr_RYMV3_R1*			Tog5307, Tog12086, Tog1260, Tog5474, Tog5286, Tog5747	Pidon et al., 2017, 2020
		NIr_RYMV3_-x			Tog5438, Tog5556	
		*NIr_RYMV3_-y*			Tog6220	

S1, serotype 1; S2, serotype 2; S3, serotype 3; S4, serotype 4; S5, serotype 5; S6, serotype 6; VPg, viral genome linked protein.

1 RB: resistance breaking isolates.

The second *S* gene, *RYMV*2, was identified in the *O. glaberrima* accession Tog7291. *RYMV2* is predicted to be localized on chromosome 1, encodes a homolog of the *Arabidopsis CONSTITUTIVE EXPRESSION OF PATHOGENESIS RELATED GENE-5-1* (*CPR5-1*) (Thiémélé et al., 2010; Orjuela et al., 2013; Pidon et al., 2020). In rice, two *Arabidopsis CPR5* homologs are reported to be present, *CPR5-1* and *CPR5-2*. Resistance conferred by *CPR5* is linked to a point or nonsense mutation in the *CPR5-1* sequence (Orjuela et al., 2013). This gene encodes a transmembrane nucleoprotein in *Arabidopsis* and is known to regulate effector-triggered immunity by controlling the cell cycle and defense mechanisms. In response to the activation of immunoreceptors, *CPR5* undergoes a conformational switch from oligomer to monomer. In this process, loss of function occurs and leads to the release of cyclin dependent kinase inhibitors and the permeabilization of the nuclear pore complex, which activates constitutive resistance to several pathogens (Gu et al., 2016). Despite resistance against pathogens, this loss of function mutants usually come with detrimental phenotypes that are undesirable for crop improvement (Bowling et al., 1997; Pidon et al., 2020). Six frameshift or truncated alleles have been identified in *O. glaberrima* accessions harboring *RYMV2* (Table 1), interestingly, none of them display undesirable traits. Their activity is attributed to a partial functional redundancy of *CPR5-1*, suggesting that this gene is not a functional homolog of *Arabidopsis CPR5*. The third major gene and the only R gene is *NLR*_RYMV3_, or *RYMV3*, found in the *O. glaberrima* accessions Tog5307 and Tog5672. *RYMV3* maps on chromosome 11 and encodes for a NB-LRR (Pidon et al., 2017, 2020). The LRR proteins represent one of the most diverse and abundant classes of R-gene families in plants and can be variable even among closely related plants due to presence or absence of polymorphism (Dodds and Rathjen, 2010; Luo et al., 2012; Tan and Wu, 2012; Marone et al., 2013). At the *RYMV3* locus, three candidate alleles have been mapped: two alleles, *Nir_RYMV3_R1* and *Nir_RYMV3_-x*, involve a substitution of one amino acid at position K779R and A823V, respectively. The resistance conferred by the third allele, *NIr_RYMV3_-y*, is a result of a truncated protein in the LRR domain with 11 amino acid substitutions (Pidon et al., 2020). The molecular basis of NB-LRR gene resistance to virus infections is expressed in two forms: first, the hypersensitivity reaction restricts the virus to the primary infection site, and second, the extreme reaction, in which the intercellular movement of the virus is completely stopped (Dodds and Rathjen, 2010). Resistance mediated by *RYMV3* displays features of an extreme reaction with no symptoms expressed after infection (Pidon et al., 2020).

Collectively high genetic diversity for resistance has been identified among *O. glaberrima* genotypes compared to the commercially important species *O. sativa* (Albar et al., 2006; Thiémélé et al., 2010; Orjuela et al., 2013; Pidon et al., 2020) (Table 1). Among *O. sativa* accessions, only one major resistance gene has been found with two resistance alleles, while three major resistance genes, each with multiple alleles have been discovered in *O. glaberrima* accessions. Recessive genes (*RYMV1* and *RYMV2*) have less polymorphisms, which is attributed to conservative selection leading to low mutation rates. For instance, five and 10 non-synonymous mutations have been identified in *RYMV1* and *RYMV2*, respectively, whereas the R gene (*RYMV3*) is highly polymorphic with about 35 non-synonymous mutations identified (Pidon et al., 2020). These high mutation rates have been observed in all LRR genes due to selective pressure, which promotes the evolution of new receptors to counteract pathogens effectors (Mondragón-Palomino et al., 2002; Marone et al., 2013). This feature might be responsible for high allelic diversity in resistance loci of *O. glaberrima* (Pidon et al., 2020). A shorter evolutionary interaction between RYMV and *O. glaberrima* is however predicted as RYMV could have emerged only a few decades (Pinel-Galzi et al., 2015; Trovao et al., 2015; Pidon et al., 2020). Thus, the high variability of *RYMV3* gene is interesting, and might confer resistance to other pathogens.

The use of genetic resistance is an early prevention method to reduce the impact of viral diseases (Todorovska et al., 2009; Marone et al., 2013). However, the deployment of novel varieties harboring major genes is affected by the ability of pathogens to rapidly evolve and overcome such resistance. Most R genes are known to be race specific and only give resistance to a single or limited strain(s) of a given a pathogen (Burdon et al., 2014). The mechanisms of RYMV evolution responsible for its genetic diversity are presumably 2-fold: mutations (N’guessan et al., 2000) and recombination (Ochola et al., 2015; Ndikumana et al., 2017). Recombination analyses found that two new isolates, Ke101 and Ke105, evolved from homologous recombination between strains S4lv and S4ug recognized as emerging types from Western Kenya (Adego et al., 2018), whereas mutations in the central domains of the VPg or different regions of polyprotein P2a contribute to emergence RYMV variants that overcome major resistance genes (Hébrard et al., 2008b, 2010, 2018; Poulicard et al., 2014; Pinel-Galzi et al., 2016; Pidon et al., 2020).

Mutations in the VPg are predicted to develop in three sequential ways (Pinel-Galzi et al., 2007; Traoré et al., 2010; Poulicard et al., 2014). Pathway I occurs *via* the sequential substitution of arginine (A) with glycine (G) and glutamic acid (E); pathway II involves sequential mutations to valine (V) but coexists with isoleucine (I); and pathway III involves sequential mutations substituting arginine with tryptophan (W). Pathway I is more frequent and efficient, whereas II and III are isolate specific (Pinel-Galzi et al., 2007; Traoré et al., 2010). In addition, polymorphism at VPg codon 49 which involve either glutamic (E) or threonic (T) acid residues (E/T polymorphism) influences the virulence of RYMV isolates (Poulicard et al., 2012). This polymorphism involves three pathotypes. The first are the T-pathotypes, in which isolates with threonine (T) at codon 49 only break resistance in *O. glaberrima* accessions. T-pathotypes with substitutions at codons 41 and 52 of the VPg overcome resistance conferred by *rymv1-3* and *rymv1-5* alleles, respectively, while substitutions from threonine to alanine at position 43 overcome resistance of the *rymv1-4* allele. The T-pathotypes with substitutions at codon 804, exchanging phenylalanine for leucine in polyprotein P2a overcome *RYMV2*-mediated resistance (Pinel-Galzi et al., 2016) and *RYMV3* encoded resistance (Pidon et al., 2017), although the mechanism of resistance breakdown is not fully understood. The second are the E-pathotypes with a glutamic acid at codon 49 which overcome *rymv1-2* resistance in *O. sativa*. These pathotypes are polymorphic at codon 48 of VPg, and in avirulent isolates, a conserved arginine is present in this region. Substitutions of R48E and E309K in the VPg of E-pathotypes and eIF(iso)4G, respectively, provide direct interaction between the viral VPg and eIF(iso)4G (Pinel-Galzi et al., 2007; Hébrard et al., 2008b, 2010; Traoré et al., 2010). The third is a highly virulent pathotype suspected to overcome all known sources of resistance. The Hypervirulent T9-pathotype contains a mutation in the central domain of the VPg and was recently identified in West-Central Africa (Hébrard et al., 2018).

Geographically, the E-pathotypes are prevalent in East Africa while the T-pathotypes are common in West Africa. Similarly, the geographic clustering of rice accessions harboring resistance alleles in *RYMV1*, *RYMV2*, and *RYMV3* loci has been recently demonstrated (Pidon et al., 2020). Thus, the geographic specificity of isolates and resistance genes clearly indicates a pattern of adaptation which would guide the development and deployment of rice breeding products in SSA. Resistance coffered by *rymv1-5* and *RYMV3* are quite durable in both regions (Hébrard et al., 2018; Pidon et al., 2020), whereas resistance mediated by *rymv* 1-3, rymv 1-4 and RYMV2 might be effective in only East Africa. However, the high durability of rymv1-4 in Tog5672 than in Tog5438 suggests epistatic control of resistance.

## Genetic Determinants and Mechanisms of Quantitative Resistance to RYMV

Quantitative disease resistance is controlled by multiple genes (minor genes) with small effects or quantitative trait loci (QTLs). Quantitative resistance is characterized by a reduced virus load in the plant tissues and a slow progression of disease development due to the weakened movement of the virus. Thus, because it does not show the dramatic breakdown observed with major R gene deployment, quantitative resistance is usually viewed as durable and consequently often used in a broad range of crops (Parlevliet, 2002; Burdon et al., 2014).

Quantitative resistance to RYMV is limited to the 1st week of infection, beyond which the disease progresses, virus titer increases, and symptoms reach levels comparable to those of susceptible varieties but with less loss of yield (Albar et al., 1998; Ioannidou et al., 2000). Quantitative resistance to RYMV has been identified mostly in tropical upland rice varieties such as Azucena and Moroberekan (*Japonica* subtypes) (Ghesquière et al., 1997; Ioannidou et al., 2003). Fifteen QTLs have been uncovered on seven chromosomal fragments. Using an Azucena × IR64 double haploid population, genetic mapping identified these QTLs on chromosomes 1, 2, 4, 7, 8, 9, and 12 (Boisnard et al., 2007). However, most of these QTLs co-localize with growth and development QTLs, except for those on chromosome 12, and are, therefore, difficult to target. The RYMV QTL on chromosome 12 is considered to be major (Ghesquière et al., 1997; Albar et al., 1998; Ioannidou et al., 2003; Boisnard et al., 2007). Epistatic interactions between QTLs on chromosomes 7 and 12 presumably play a vital role in the regulation of partial resistance to RYMV in Azucena (Pressoir et al., 1998; Ahmadi et al., 2001; Ioannidou et al., 2003), which might limit their usefulness in marker assisted selection (MAS) taken into consideration. Other partial resistance QTLs are probably in similar positions with the major RYMV resistance genes (Boisnard et al., 2007). For example, Orjuela et al. (2013) reported on the co-localization of QTL1 in a 151-kb interval of the RYMV2 gene, hence this QTL may combine complete and partial resistance. Genome-wide association analysis study (GWAS) involving various accessions of *O. glaberrima* also revealed considerable relationships between quantitative resistance and two known major resistance genes (Cubry et al., 2020). In the same study, several single nucleotide polymorphisms (SNPs) close to two major resistance genes *RYMV1* and *RYMV3* were identified. However, the SNPs associated with resistance conferred by *RYMV1* and *RYMV3* were not found in regions flanking any of the identified partial resistance QTLs indicating differences in genes and pathways of resistance (Cubry et al., 2020) suggesting the complex mechanism of partial resistance to RYMV in rice.

## Breeding for RYMV Durable Resistance

Breeding for disease resistance has been the most sustainable way of crop improvement to avert potential crop yield losses due to plant viruses (Burdon et al., 2014; Mundt, 2014; Fuchs, 2017). Genetic resistance is durable if it is effective over time when deployed in an environment that is favorable for disease development (Johnson, 1981; Kobayashi et al., 2014). Resistance durability of a gene depends on the nature of resistance, pathogen variability, and environmental factors (Maule et al., 2007; Gómez et al., 2009; Fonseca and Mysore, 2019). These factors presumably influence the epidemiology of RYMV and complicate the deployment of control strategies across Africa (Traoré et al., 2009; Trovao et al., 2015).

The context of durable resistance depends on a given breeding programs, for instance, lasting resistance is critical when new varieties are released less frequently compared to programs that often release novel resistant varieties (Nelson et al., 2018). Generation of broad-spectrum resistance has been the focus of most breeding programs for RYMV resistance. Preliminary focus of breeding for RYMV resistance has been on the deployment of resistance derived from Gigante (*rymv1-2*), given its *O. sativa* background and high F1 hybrid fertility in cross combinations. Recently, rymv1-2 allele was introduced into some elite cultivars, and several near-isogenic rice lines (NILs) were generated (Ndjiondjop et al., 2013). These NILs combine the rymv1-2 allele and agronomically important traits. Efforts to pyramid RYMV major resistance genes and resistance QTLs through conventional breeding methods have proven futile (Ndjiondjop et al., 2013). This has been attributed to the recessive nature of resistance conferred by most RYMV genes. If a recessive gene is introduced into an elite variety by backcrossing, the theoretical segregation ratio between the heterozygotes with recessive resistance and homozygotes with no resistance gene is 1:1 in the first generation (Griffiths et al., 2012). At this stage, it is difficult to discriminate which individuals are the heterozygotes. The heterozygotes must be tested in the second generation, which requires more time and resources. However, the use of marker assisted breeding (MAB) would allow effective selection of rymv recessive alleles in the heterozygous state. No selfing or test crossing is needed to detect rymv alleles in populations, thus saving time and accelerating breeding progress.

The use of MAB would also provide opportunities to combine R and or S genes with QTLs that could provide durable and more resilient forms of resistance to RYMV. Thus, it is important to understand the impact of each of the RYMV major resistance genes alone or in combination with other resistance genes in the field before deployment. Combining the candidate QTLs with RYMV major resistance genes in a single genetic background would substantially enhance the durability of resistance as the partial resistance would delay the breakdown of the major resistance gene (Mundt, 2014; Fuchs, 2017). Thus, more QTLs for partial resistance should be identified and included in the breeding program in SSA to enhance the durability of *rymv1-2* and other major genes. Additional ways to improve durability of a major resistance gene is to combine them with another major gene (Rimbaud et al., 2018), especially those that interrupt the interaction with the conserved domains of the virus genome during the infection cycle. This would reduce the genetic adaptation of virulent variants to their environments and hosts (Fuchs, 2017). Pyramiding the recessive (*RYMV1*) and the dominant (*RYMV3*) resistance genes may provide much desired broad-spectrum resistance against RYMV and other pathogens in rice.

## Integration of New Breeding Techniques for RYMV Resistance

The continuous improvements in genomics and bioinformatics has a potential to speed up crop breeding (Batley and Edwards, 2016). Advances in genomic-assisted selection have become essential to facilitate the introduction of traits that may not be done using conventional breeding techniques. Molecular markers can be used to facilitate the selection of rare traits at early stages without phenotypic analysis, thus speeding up plant breeding and crop improvement (Varshney et al., 2005; Batley and Edwards, 2016).

The scope of MAS breeding for targeted introgression of RYMV resistance genes has been successfully demonstrated in SSA (Ndjiondjop et al., 2013). A number of SNP markers linked to *rymv1-2*, *rymv1-3*, *rymv1-4*, and *rymv1-5* alleles are now available (Albar et al., 2003; Thiémélé et al., 2010). Improvements in high-throughput genotyping have enhanced the identification of QTLs through GWAS. For instance, Cubry et al. (2020) observed a total of 2,199 SNPs, close to chromosomes 4 and 11 associated with *RYMV1* and *RYMV3* resistance, respectively. However, adoption and utilization of genomics-selection approaches in many breeding programs has remained low due to less know-how on genomic tools, and the cost involved in MAS (Ndjiondjop et al., 2013). However, given the current developments in refining SNPs associated with all RYMV major resistance genes at the International Rice Research Institute (IRRI), Institut de Recherche pour le Développement (IRD), and AfricaRice, it is likely that introgression of these genes through MAS will be more successful. These genes could also be pyramided with other biotic and abiotic stress resistance genes using anther culture techniques. Recently, successful crosses between *O. glaberrima* and Milyang23 (*O. sativa*) for resistance to multiple pathogens, such as RYMV, Bacterial leaf blight, Rice blast and Bacterial leaf streak, was demonstrated using anther culture techniques. Hybrids with combined resistance to these diseases were shown to be highly resistant to all four pathogens (Lamo et al., 2015).

Genetic engineering is another important strategy in crop improvement against plant viruses, considering the limited sources of natural resistance and the constraints related to the introduction of these genes to popular cultivars. RNA interference (RNAi) is one of the methods that has been explored to develop transgenic virus-resistant rice (Pinto et al., 1999; Sasaya et al., 2014; Kreuze and Valkonen, 2017). This approach involves the expression of hairpin RNA constructs in the host plant (Sanford and Johnston, 1985; Baulcombe, 1996; Brodersen and Voinnet, 2006), and it has shown success in generating RYMV resistant transgenic plants targeting the conserved P2a protein in the susceptible cultivar IR64 (Pinto et al., 1999). Additionally, genome editing is another approach with enormous potential to improve breeding for RYMV resistance. Genome editing tools, such as CRISPR-Cas9, are powerful techniques to facilitate crop improvement. CRISPR-Cas9 is based on introducing targeted mutations at specific sites by utilizing non-homologous end-joining to repair induced double-strand breaks (Belhaj et al., 2013; Haque et al., 2018; Kalinina et al., 2020). Full implementation of CRISPR-Cas9 technology is dependent on the identification of plant host factors which can be manipulated to limit virus proliferation (Kalinina et al., 2020). Some of these host factors have been demonstrated to interact directly with the viral RNA (Hyodo et al., 2014; Van Schie and Takken 2014; Garcia-Ruiz, 2018), although only a few of them have been identified to date. In rice, only *eIF(iso)G4*, *CPR-5* (Albar et al., 2006; Thiémélé et al., 2010), is known to interact with RYMV. This limited number of plant-interacting proteins, with experimental evidence, makes targeting specific host genes for gene modification difficult. Therefore, to provide more breeding options for RYMV resistance, additional susceptibility genes need to be identified and validated. Once validated, these genes can be modified using CRISPR-Cas9 to disrupt the interaction with viral proteins. This brings the susceptible host plant outside the host range of the virus, to confer resistance.

## Conclusion

RYMV continues to cause losses to rice production in SSA due to lack of resistant elite cultivars. Considering the high adaptive potential of this virus and frequent resistance breakdown in rice cultivars, breeding for resistance and its durability should be given top priority in SSA. Encouraging news comes from recent knowledge on rice-RYMV interactions, which has enabled identification and isolation of three major resistance genes (*RYMV1*, *RYMV2*, and *RYMV3*), and the subsequent definition of geographic specificities of these genes and RYMV strains. There is need to build on these successes by further identifying the genomic localization of desired SNPs linked to the major resistance genes for use in genomic-assisted breeding. The speedy development of homozygous lines through double haploid breeding when coupled with MAS would be more effective for developing and deployment of durable RYMV resistant varieties. Parallel efforts are needed to identify additional genes and extensively screen host-virus protein interactors to identify and validate additional host factors that assist or suppress the virus. With these interaction factors identified, a reverse genetics approach could be used to identify novel host *S* genes that could be modified by genome editing to impair susceptibility. These short and long-term strategies will provide immediate products and build a large germplasm base and knowledge necessary to respond to future RYMV attacks.

## Author Contributions

PO wrote the first draft of the manuscript. GO helped to write and edited the manuscript. PO and GO drew the figures and tables. KG edited, supervised, and supported the writing. KN, TA, and OR edited all versions of the manuscript. All authors contributed to the article and approved the submitted version.

### Conflict of Interest

The authors declare that the research was conducted in the absence of any commercial or financial relationships that could be construed as a potential conflict of interest.
